# Marvellous moths! pollen deposition rate of bramble (*Rubus futicosus* L. agg.) is greater at night than day

**DOI:** 10.1371/journal.pone.0281810

**Published:** 2023-03-29

**Authors:** Max Anderson, Ellen L. Rotheray, Fiona Mathews

**Affiliations:** School of Life Sciences, University of Sussex, Falmer, Brighton, United Kingdom; Universidade Federal de Uberlandia - Campus Umuarama, BRAZIL

## Abstract

Widespread concerns about declines of wild pollinating insects has attracted considerable research interest, largely directed towards identifying key nectar sources and assessing the contribution of pollinators towards ecosystem services. However, previous work has almost exclusively focussed on bees and other diurnal invertebrate taxa. This study aimed to assess the relative contribution of diurnal and nocturnal insects to the pollination of bramble (*Rubus fruticosus* agg.), a common and widespread species aggregate across Europe, which has been identified as a key source of pollen and nectar for diurnal pollinators. Bramble flower visitation rates by diurnal and nocturnal insects were quantified by analysing over 380,000 interval photographs taken over a 3-day period across 10 sites. A pollinator exclusion experiment was also undertaken to assess the importance of diurnal and nocturnal insects for pollination by analysing pollen deposition on 480 bramble stigmas of nocturnally exposed, diurnally exposed and unvisited control flowers across all sites. Flower visitation was significantly higher during the day, comprising 83% of all visits made by a range of taxa. Nocturnal visitation was almost exclusively by moths. Crucially, pollen deposition rates of bramble were significantly higher during the night compared with the day. No relationship was detected between pollen counts and flower visitation rates, suggesting that moths are more efficient pollinators of bramble compared with diurnal insects. Overall, this work provides further evidence of the value of bramble as a resource for pollinators and demonstrates that moths likely play an important role in bramble pollination.

## Introduction

Pollinating insects are vital components of many ecological communities, where they contribute towards pollination of a diverse array of ecologically and economically important crops and wild plants [1,2]. Wild pollinators are experiencing widespread declines, largely attributed to agricultural intensification and climate change [3,4]. Within agricultural ecosystems, scrub forms a key component of a number of terrestrial habitats of principal importance for the conservation of biodiversity [5]. Bramble, *Rubus fruticosus* L. aggregate (Rosaceae) is an aggregate group of around 300 microspecies of fruiting, perennial shrubs native to Europe [6], and is characteristic of many scrub habitats [7]. The flowering period of bramble is typically from May to October, and ripe fruit is produced from late summer through to autumn [8]. Previous work has established that the volume of nectar produced by bramble is greatest during the early morning and late evening compared with midday, and nectar sugar concentrations are higher in the evenings [9]. Bramble has a potentially important role to play in conserving threatened and declining taxa. Not only does it serve as a refuge and nesting site for birds and mammals [10–12], but the long flowering period also provides an key source of pollen and nectar for bees, hoverflies, butterflies and many other diurnal invertebrates throughout the summer and into autumn, when nectar sources are more limited [13]. However, very little is known about the contribution of nocturnal pollinators, with almost all pollination research having been conducted on diurnally-active insects [14].

Moths are a highly diverse group of invertebrates that are considered to the main contributors to nocturnal pollination [15]. Despite moths comprising 88–91% of all described Lepidoptera, they are subject to a disproportionately low level of research compared with butterflies [16]. In addition to facing many of the same pressures as their diurnal relatives, moths are also threatened by factors that are unique to a nocturnal lifestyle e.g. artificial light at night [17–20]. As a consequence, many macro moths have experienced significant declines in Britain, with a 33% reduction in total abundance between 1968–2017, and many of the declines particularly affecting bioabundant species [21].

This study assesses the contribution of nocturnal and diurnal invertebrates to the pollination of bramble by monitoring visitation of flowers. In addition, a pollinator exclusion experiment was also conducted to assess the importance of insects in the pollen transfer on bramble (*Rubus fruticosus* agg.) flowers and to compare the contribution of nocturnal and diurnal pollinators.

## Materials & methods

### Site selection

Ten sites in the south-east of England were selected for inclusion based on their implementation of low to moderate intensity grazing with a specific view to maintain or enhance biodiversity, and by the presence of bramble in abundance at the site. Flower visitation and pollen deposition of bramble were sampled simultaneously and independent of each other over a period of three consecutive days and nights between 4^th^ July– 24^th^ July 2021 (S1 Table). All sites were also within 30km of each other to minimise any variation owing to geography or weather. Sampling was undertaken when mean day and night temperatures exceeding 17°C and 12°C respectively, and with mean daily rainfall below 1mm (S1 Table). Grazing livestock were present in all sites during the sampling period.

### Flower visitation

The number of bramble flower-visiting invertebrates was determined by taking interval photographs of flowers during the day and night. Six Victure HC100 wildlife cameras (16-megapixel) with infrared low glow technology, were set to take photographs every 30 seconds over a period of three days and three nights at each site. Cameras were inactive between 16:00hrs and 19:30hrs each day to maximise battery life over the sampling period and to ensure that visitation was captured around sunrise and sunset periods (S2 Table). Cameras were secured to 1.5m wooden stakes and were focussed on an individual inflorescence with multiple open flowers that was within 1 to 2 metres of the camera.

Images from the cameras were exported and manually processed to identify those with invertebrate visitors. For each camera trap, between two and ten anthesised (open) flowers were observed to count the number of insect visitors. Any image where an invertebrate is clearly in contact with the flower was classed as ‘visited’. Due to limitations on the resolution of images collected, only macroinvertebrates were identified. *Apis mellifera* and solitary bees were largely indistinguishable as a result of the camera quality and so these two groups were combined to avoid any misidentification. Other invertebrates were identified as being: *Bombus spp*., Diptera (*Syrphidae*), Diptera (other), Coleoptera, butterflies and moths. Any uncertain identifications were labelled as ‘no ID’. The total number of visiting invertebrates during the day and night was calculated and adjusted to give a total count of visits (flower^-1^) over the three-day period, hereafter referred to as ‘total visits’. Day and night lengths were obtained by calculating the difference between sunset and sunrise times. The upper and lower 95% confidence intervals for percentage visitation of each species group at day and night was calculated by using the Wilson score interval. To account for the differing numbers of hours available for insects to visit at night and in the day, the rate at which insects visited was calculated (flower^-1^ hour^-1^), hereafter referred to as ‘visit rate’.

### Pollen deposition

Buds of bramble were bagged prior to anthesis using muslin bags (8 x 12cm) to prevent any visitation by pollinators, and were allocated to one of three treatment groups: (1) Diurnal, where bags were removed at sunrise and replaced at sunset (S2 Table), so that flowers were exposed during the day, (2) Nocturnal, where bags were removed and replaced so that flowers were exposed from sunrise to sunset, and (3) Unvisited control, where flowers remained bagged throughout the sampling period to exclude any visiting insects. At each site, 24 bags were deployed in clustered groups of three, with one bag per treatment group. Bags were tightly secured to the branch using drawstrings in order to prevent any organisms from visiting the bagged flowers. Following anthesis, visits were made within 30 minutes of sunrise and sunset to remove and replace bags for the nocturnal and diurnal treatment groups for a total sampling period of three days and three nights. Sampling of pollen deposition was independent of the flower visitation experiment, though they both took place simultaneously.

Following the three day and night sampling period, flowers from each bag were carefully removed and stigmas from the centre of the pistil were extracted using forceps, avoiding making contact with anthers. Stigmas were preserved in 100% ethanol in a 1.5ml microtube before being processed in the laboratory. For each 1.5ml microtube, two stigmas were randomly selected and mounted separately, following methods described by Kearns and Inouye [22], with 16 samples per treatment group and a total of 48 samples per site. The total number of all pollen grains within each slide was counted manually using a Euromex StereoBlue compound microscope. The total number of pollen grains was determined for each stigma (‘total pollen’), and the hourly rate of pollen grain deposition (‘pollen rate’) was calculated for the nocturnally and diurnally exposed flowers by dividing the total pollen by the amount of time flowers in each treatment group were exposed for.

### Statistical analyses

All statistical analyses were undertaken using R version 4.1.1 [23]. In order to assess whether there was any difference in visitation rates of bramble flowers during the day and night, generalised linear mixed effects models were constructed with a negative binomial error distribution using the ‘lme4’ package [24]. Total visits was set as the dependent variable, daylight period (diurnal = 0; nocturnal = 1) and sampling date were specified as fixed effects, with site number as a random effect to account for autocorrelation of replicates within each site. In order to account for the differences in the number of flowers that were monitored for visitation, and the differences in the time spent recording flower visitation during the day and night, offset variables were included for the number of hours of recording and number of flowers monitored (a spike of flowers naturally contained variable numbers).

Secondly, to investigate any differences in the total amount of pollen deposition between the three treatment groups (unexposed control = 0; diurnally exposed = 1; nocturnally exposed = 2), generalised linear mixed effects models were built, using a negative binomial error distribution. Total pollen was set as the dependent variable, with treatment group and sampling date specified as fixed effects, and site number as a random effect.

To investigate the relationship between pollen deposition rates and flower visitation rates, generalised linear mixed effects models were constructed, using a negative binomial error distribution. Total pollen count was set as the dependent variable, with treatment group (diurnally exposed = 0; nocturnally exposed = 1), sampling date and total number of flower visits as fixed effects, and site number as a random effect. In order to account for differences in the amount of time that the different treatment groups were bagged, an offset variable was included for the amount of time flowers were exposed for. To test whether any relationships between pollen rate and visitation rate varied according to the nocturnal or diurnal period, an interaction term was included. For the interaction term, stepwise removal was undertaken and subsequent models were tested using analysis of variance (ANOVA). Where interaction terms were significant (*p* < 0.05), further analyses were conducted on the nocturnal and diurnal treatment groups separately.

Finally, to determine whether the pollen deposition rates depended on the visitation of specific insect groups, separate generalised linear mixed effects models were created for the pollen deposition rates of the diurnal and nocturnal treatment groups, using negative binomial distributions (because the insect groups active at night are largely non-overlapping with those that are diurnally active). For the set of models relating to the diurnal treatment group, total pollen count was set as the dependent variable, with sampling date and total number of flower visits by *Diptera* (*Syrphidae*), *Diptera* (other), *Apis mellifera*/solitary bees and *Bombus spp*. set as fixed effects, and site number as a random effect. For the models relating to the nocturnal treatment group, the same model structure was used but with visitation rates of moths, rather than other genera, set as a fixed effects. For all models, an offset variable was included for the amount of time flowers were exposed for, in order to account for differences in the amount of time that the different treatment groups were bagged for.

Model selection involved a step-wise removal approach, starting from a global model containing all variables. Prior to the step-wise removal approach, models were fitted with the most appropriate error structure, as determined by comparisons of model AIC and over-dispersion ratios. For step-wise removal, the least significant predictor variables were removed one at a time and comparisons between subsequent models were made using analysis of variance (ANOVA, Chi-squared distribution), and again, checking for over-dispersion and comparing model AIC. If the ANOVA revealed no significant differences, over-dispersion ratios were reasonably close to 1 and AIC values were similar between models, then the most parsimonious model, was selected. Checks for collinearity of predictor variables were also undertaken by generating Variance inflation factor (VIF) values, using the ‘car’ package [25]. No predictors were removed as a result of VIF analyses, with all values being < 3. Tests of pairwise differences between levels of any significant fixed effects were conducted using Tukey adjusted post-hoc tests using the ‘lsmeans’ package [26].

## Results

### Visitation of flowers

A total of 389,677 images were collected across all sites during the 3-day sampling periods (S3–S6 Figs). There were 11,564 recorded insect flower-visiting events, and the most frequently recorded flower-visiting insects were Diptera (*Syrphidae*) (55% of visits per flower), followed by moths (16%), *Bombus spp*. (8%), Diptera (other) (8%) and *Apis mellifera*/solitary bees (7%) (Table 1).

**Table 1:** Total number of visits (flower^-1^) for each insect group across all sites.

Taxonomic group	Total visits (flower^-1^)	
	Diurnal	Nocturnal
	n (%)	n (%)
**Diptera (*Syrphidae*)**	1839.44 (51.1%, 58.7%)	5.87 (0%, 11.2%)
***Bombus spp*.**	261.76 (0%, 17.8%)	0
**Diptera (other)**	255.55 (2.3%, 12.9%)	3.53 (0%, 11.0%)
***Apis mellifera* / solitary bee**	221.58 (0%, 17.1%)	0.33 (0%, 5.3%)
**Lepidoptera (butterfly)**	180.97 (0%, 14.2%)	0
**Lepidoptera (moth)**	20 (0%, 6.6%)	547.73 (8.3%, 24.3%)
**Coleoptera**	1.17 (0%, 5.3%)	0
**No ID**	14.76 (0%, 11.3%)	0
**Total**	2795.2 (83.4%)	557.5 (16.6%)
**Grand total**	3352.7	

Visitation counts are separated by time period (diurnal and nocturnal), with the percentage of the total nocturnal and diurnal visitation count for each species group represented by lower and upper 95% confidence intervals, calculated using the Wilson score interval.

**Fig 1:**
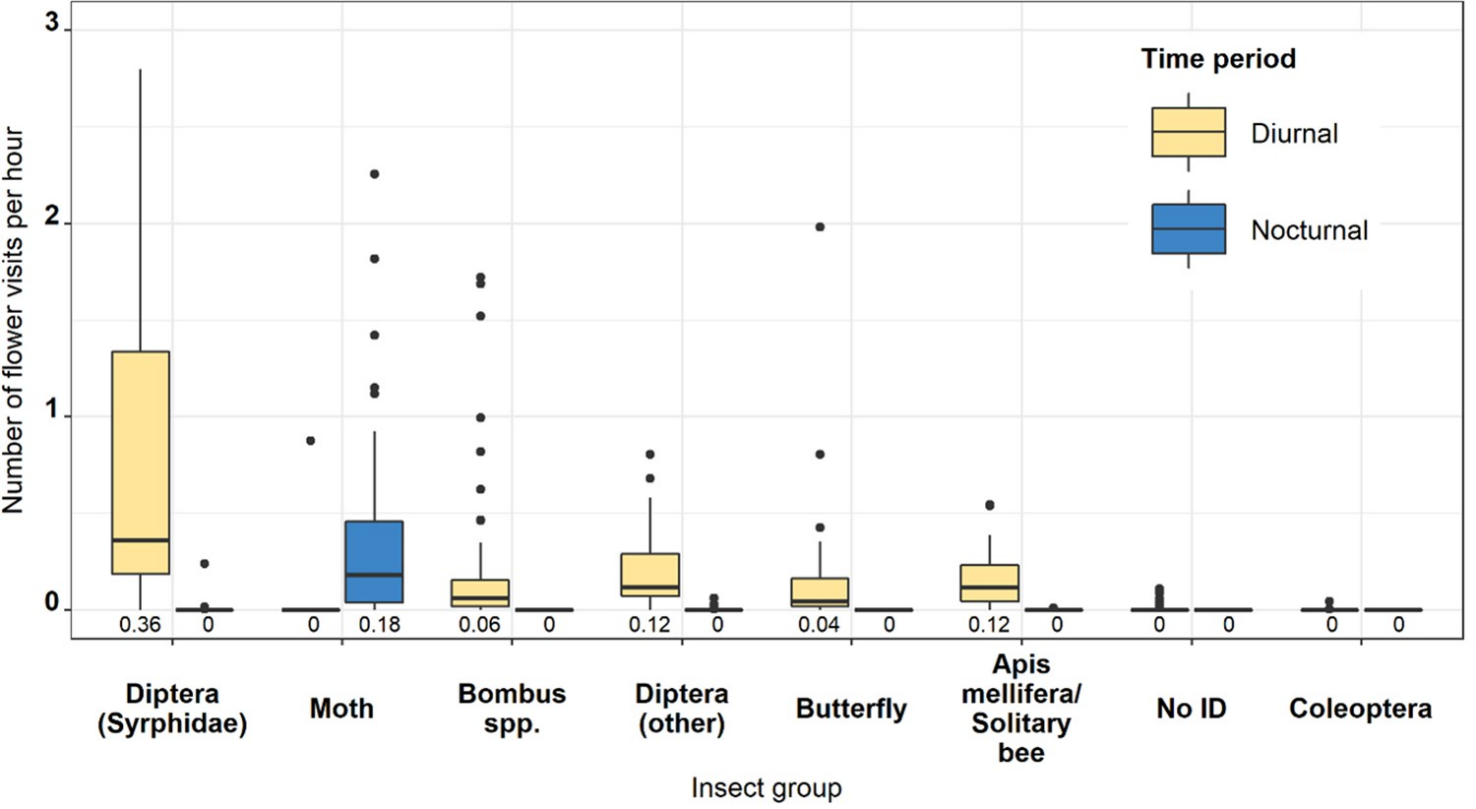
Boxplot showing the number of flower visits (hour^-1^) for each insect taxonomic group during the nocturnal and diurnal time periods. Black dots represent outlying data points and median values are presented beneath each plot.

All flower-visiting taxonomic groups recorded in this study were found to visit flowers in the diurnal period with the highest rate of visitation by Diptera (*Syrphidae*) followed by Diptera (other), *Apis mellifera*/solitary bees, *Bombus spp*. and butterflies. Visits during the nocturnal period were dominated by moths, with very rare occurrences of *Apis mellifera/*solitary bees, Diptera (*Syrphidae*) and Diptera (other) (Fig 1).

**Fig 2:**
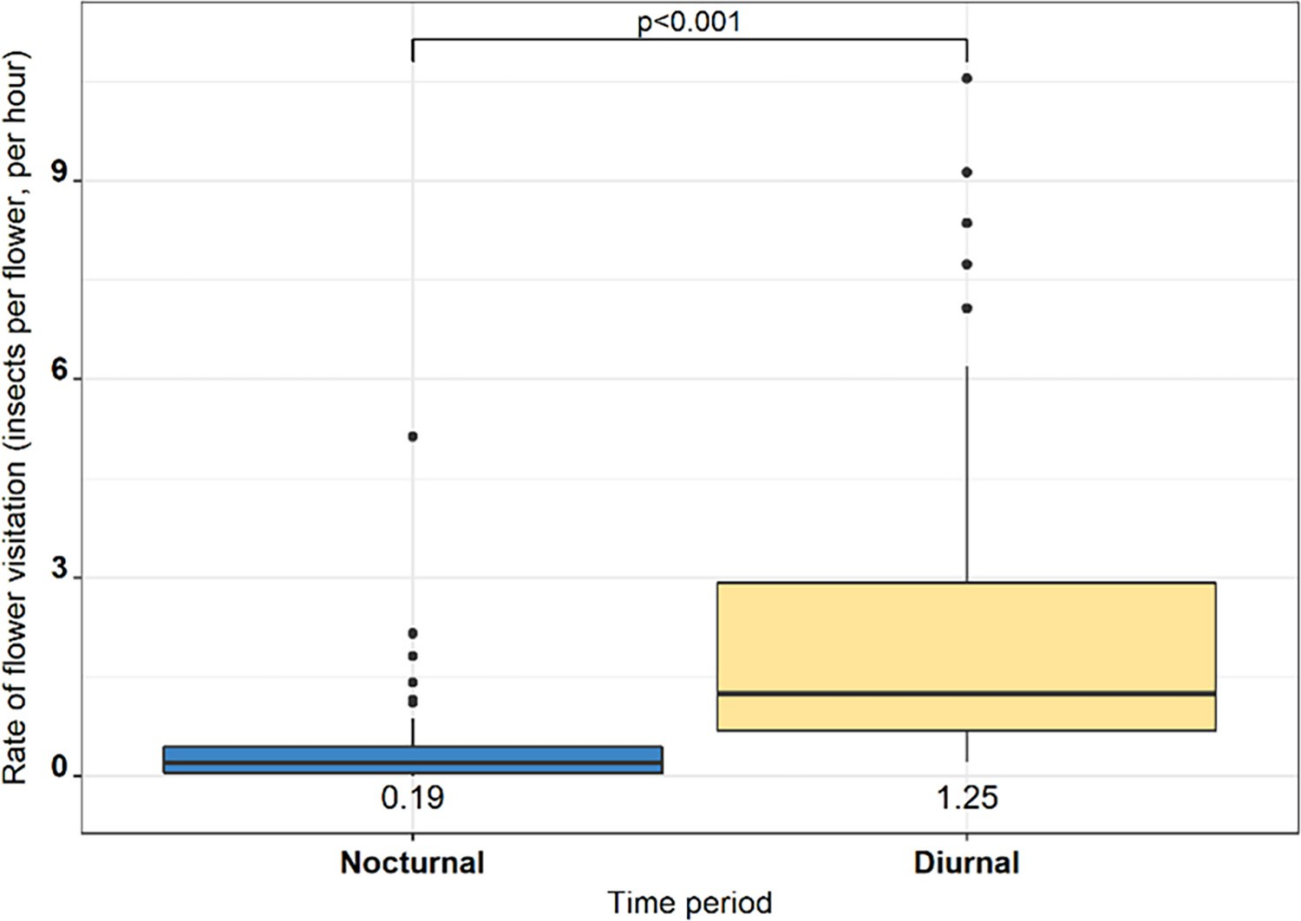
Box plot comparing the rate of flower visitation by all insects (flower^-1^ hour^-1^) between nocturnal and diurnal time periods. Black dots represent outlying data points with median values presented beneath each plot.

Bramble visitation rates (flower^-1^ hour^-1^) were significantly lower during the night compared with the day (*p* < 0.001) (Odds Ratio (OR): 0.13, 95% Confidence Intervals (CI): 0.08–0.22) (Fig 2). There were no significant differences in rate of flower visitation between the three sampling dates.

### Pollen deposition

**Fig 3:**
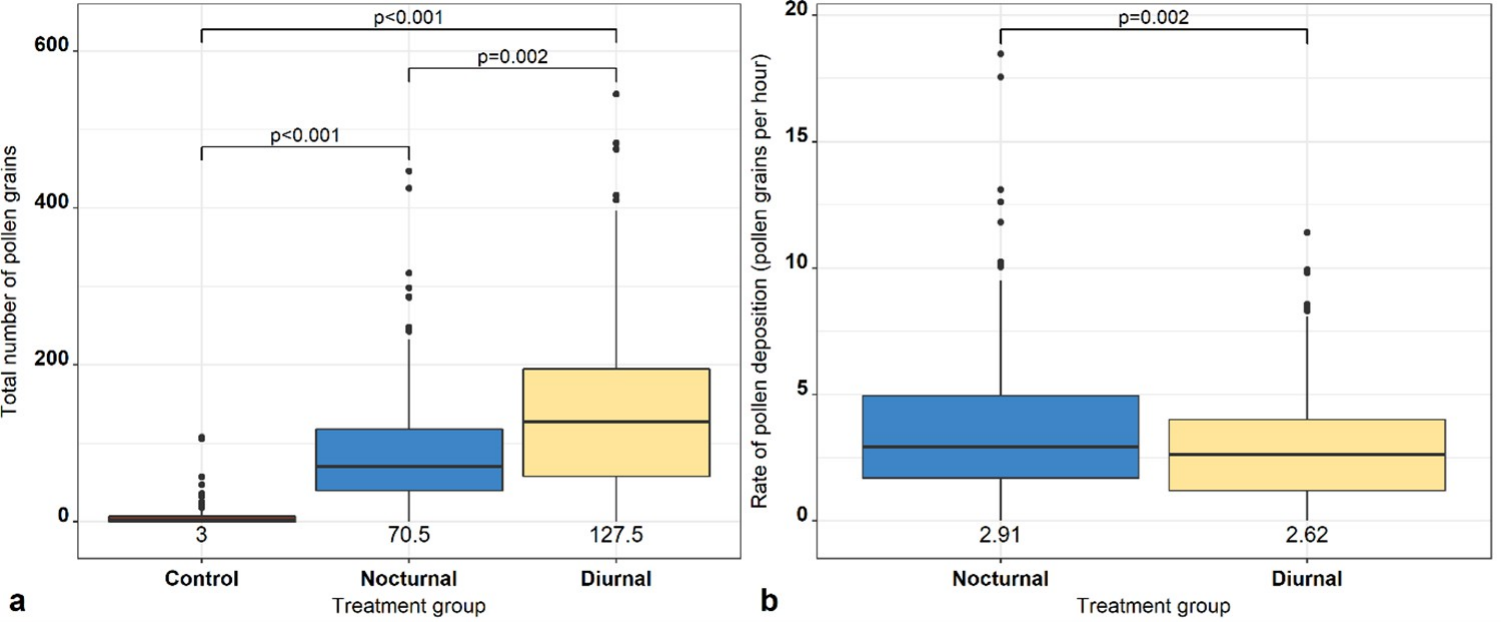
Box plots comparing a) total number of pollen grains deposited between the three treatment groups (unvisited control, nocturnally exposed and diurnally exposed flowers), and b) the rate of pollen deposition (hour^-1^) between the nocturnally exposed and diurnally exposed flowers. Black dots represent outlying data points and median values presented beneath each plot.

Across all sites, a total of 38,216 pollen grains were counted. There were significant differences in the total amount of pollen deposited between treatment groups, with more pollen deposited in the diurnal (*p* < 0.001) (OR: 20.26, CI: 15.99–25.66) and nocturnal treatment groups (p<0.001) (OR: 12.74, CI: 10.06–16.14) compared with the control group. *Post-hoc* tests revealed that total number of pollen grains on control stigmas was significantly lower than those on the stigmas of diurnally exposed (*Χ*^2^ = -3.01, *p* < 0.001), and nocturnally exposed flowers (*Χ*^2^ = -2.55, *p* < 0.001), and total number of pollen grains was greater in on stigmas of diurnally exposed compared with nocturnally exposed flowers (*Χ*^2^ = 0.46, *p* = 0.002) (Fig 3A). There was no significant variation in the total number of pollen grains between sampling dates.

Using the subsets of data relating to pollen deposition for only the diurnal and nocturnal treatment groups, we found that there was no interaction between treatment group and total flower visitation rates (*p* = 0.595). However, the total pollen deposition rate (hour^-1^) was higher in the nocturnally exposed flowers compared with diurnally exposed flowers (*Χ*^2^ = 0.24, *p* = 0.002) (Fig 3B).

There were no significant relationships between pollen deposition rates and time of sampling or flower visitation rates of all insects combined. When analysing the diurnal and nocturnal periods separately, there were no significant relationships between pollen deposition rates and the visitation rates of any of the invertebrate taxonomic groups individually.

## Discussion

This study presents new evidence to show that the rate of pollen deposition for bramble is higher during the nocturnal phase than it is during the diurnal phase in low intensity grazed habitats, highlighting the potential importance of nocturnal insects for pollination of bramble. Bramble is known to be apomictic and partly pseudogamous, where individuals are able to reproduce asexually and occasionally in the absence of pollen [27]. This mechanism provides assurance of reproduction for plants that are isolated, with a genetically identical clone being produced as a consequence. In the presence of insect pollinators, it is likely that there will be an increase in genetic diversity of bramble through higher incidences of cross-pollination. While there is evidence to show that bramble fruits form in similar abundance in the presence or absence of visiting insects [28], the average size and mass of fruit developing from flowers pollinated by insects are greater compared with unvisited flowers [27]. Furthermore, previous work has also demonstrated the importance of higher diversity of visiting insects for bramble pollination and fruit-set. Coates, Brown [29] showed that the volume of bramble fruits was greater in single visited flowers compared to those that excluded visitation, and open pollinated flowers, visited by multiple pollinator species produced fruits that were around double the volume of those resulting from single visited flowers. The importance of insect pollination to the size and quality of fruits has also been demonstrated in a range of other species including apples [30], strawberries [31] and raspberries [32].

Nocturnal pollination is understudied compared to diurnal pollination [14], partly as a consequence of the logistical challenges associated with undertaking ecological fieldwork during the night. In this study, the vast majority of nocturnal flower visits (98%) were undertaken by adult macro moths, which are primarily nectar feeders [33]. Bramble produces higher volumes of nectar in the evenings compared with mornings and afternoons [9], which may drive nocturnal visitation rates. However, it is important to note that counts of pollen deposition in this study are not species-specific. Future research should therefore include a longer follow-up period in order to assess fruit set and development. Nonetheless, moths have been shown to pollinate and transport pollen from wide variety of plant species [15,19]. This work suggests that further research is needed to evaluate the role of moths and other nocturnal insects in pollinating other fruits, both wild and domesticated. The study was conducted at a time of year when the difference in day and night length is at its highest in Northern Europe (approximately 2.1:1 hours of daylight *vs*. night). The higher efficiency of moths in terms of pollen deposition rate could imply a greater impact at lower latitudes, and at other times of the year, when differences in night- and day-lengths are less pronounced.

The total amount of pollen deposited on virgin bramble stigmas in the unvisited ‘control’ flowers was substantially lower than that of the diurnally and nocturnally exposed flowers, indicating that insects contribute substantially to the deposition of pollen on virgin bramble stigmas. The importance of flower visitation by diurnally active insects on bramble pollination has been demonstrated previously [13,27]. However, we found no relationship between pollen deposition rates and visitation rates of all insects and individual insect groups. The lack of such a relationship may suggest that some visitors do not contribute towards pollen deposition or that pollination efficacy varies between species [34]. Lepidoptera spend longer at flowers compared to Diptera, both of which have substantially longer flower handling times compared to Hymenoptera [35]. Using interval photography does not allow for any determination of the time spent during a single visit, which may also have an influence on pollen deposition. While the rates of visitation and pollen deposition recorded in this study indicates that nocturnal flower visitors are more efficient at pollen transfer, the specific mechanisms driving the differences in pollination efficiency between the nocturnal and diurnal phases are unclear. Further research should explore species-specific interactions with flowers and to establish whether there are any relationships between the time individual insects spent visiting flowers (as opposed to visit rates) and pollen deposition rates.

Throughout the sampling period, bramble flowers were visited by a wide range of insect groups, providing further evidence in support of the valuable role that bramble plays as a foraging resource for invertebrates [9,36]. High diversity of visiting insects is partly as a consequence of bramble having flowers with a generalist morphology, where pollen and nectar are accessible to a wide range of species [6]. The invertebrate communities associated with bramble flower visitation between the two time periods were starkly different, with very little overlap in the groups of species recorded visiting flowers during the day and night. While nocturnal visitation was almost exclusively made by moths, during the day, there was greater diversity in visitation, with the majority of flower visits made by Diptera (*Syrphidae*). The greater frequency and diversity of visiting insects observed during the day may be explained by the daily pattern of nectar production, as bramble flowers secrete greater volumes of nectar during the day compared with the night [37]. Previous studies exploring the diurnal visitation of bramble identified the same insect groups as visitors of bramble flowers [13], with differences in the most frequently recorded visiting species attributable to the sampling methodologies applied.

Generalist plants and invertebrates are integral to the development and maintenance of pollination networks, as they provide accessible foraging resources and abundant pollination functions and services [38]. Increasing the availability of nectar and pollen sources through habitat restoration and changes to grazing management systems can not only contribute towards conservation of generalist and specialist nocturnal and diurnal insects, but may also enable the pollination of threatened plants and valuable crops through increased ecosystem function [3,39]. The implementation of lower intensity grazing regimes can support the development of scrub patches, creating habitat and structural biodiversity, which benefits moths and many other important taxa [40].

Overall, bramble is a valuable foraging resource for invertebrates with a wide range of taxa visiting flowers and most visits occurring during the day. Pollen deposition on virgin bramble stigmas occurs at a higher rate during the nocturnal phase compared with the diurnal phase, with moths making up the vast majority of nocturnal flower visits. This work indicates that moths may play an important role in pollination of brambles, and the results provide further evidence to suggest that scrub plays an important role in the provision of valuable foraging resources for diurnal and nocturnal pollinators.

## Supporting information

S1 Fig Camera trap image of a Bombus sp. visiting a bramble flower. (TIF)

S2 Fig Camera trap image of a butterfly visiting a bramble flower. (TIF)

S3 Fig Camera trap image of a moth visiting a bramble flower. (TIF)

S4 Fig Camera trap image of a moth visiting a bramble flower. (TIF)

S5 Fig (TIF)

S6 Fig (TIF)

S1 Table List of sampling dates for each of the 10 study sites with mean daily and nightly temperatures and rainfall. (DOCX)

S2 Table List of sunrise and sunset times for the sampling dates. (DOCX)
