# Supplementary figures and images for: Marvellous moths! pollen deposition rate of bramble (Rubus futicosus L. agg.) is greater at night than day
Source: PLoS One. 2023 Mar 29;18(3):e0281810. doi: 10.1371/journal.pone.0281810 (PMC10057810; doi:10.1371/journal.pone.0281810)

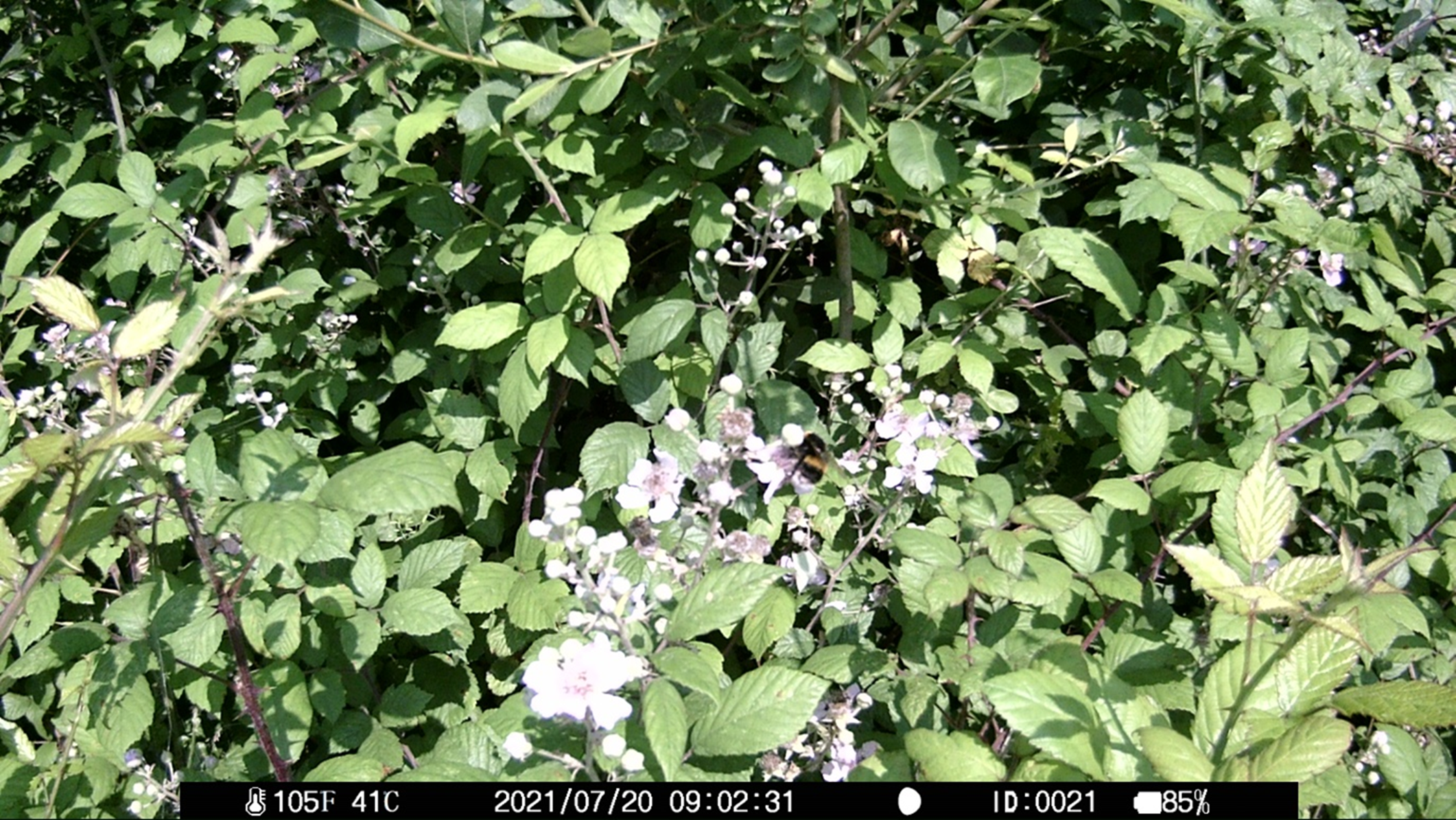

Supplement: S1 Fig — (TIF) [file pone.0281810.s001.tif]

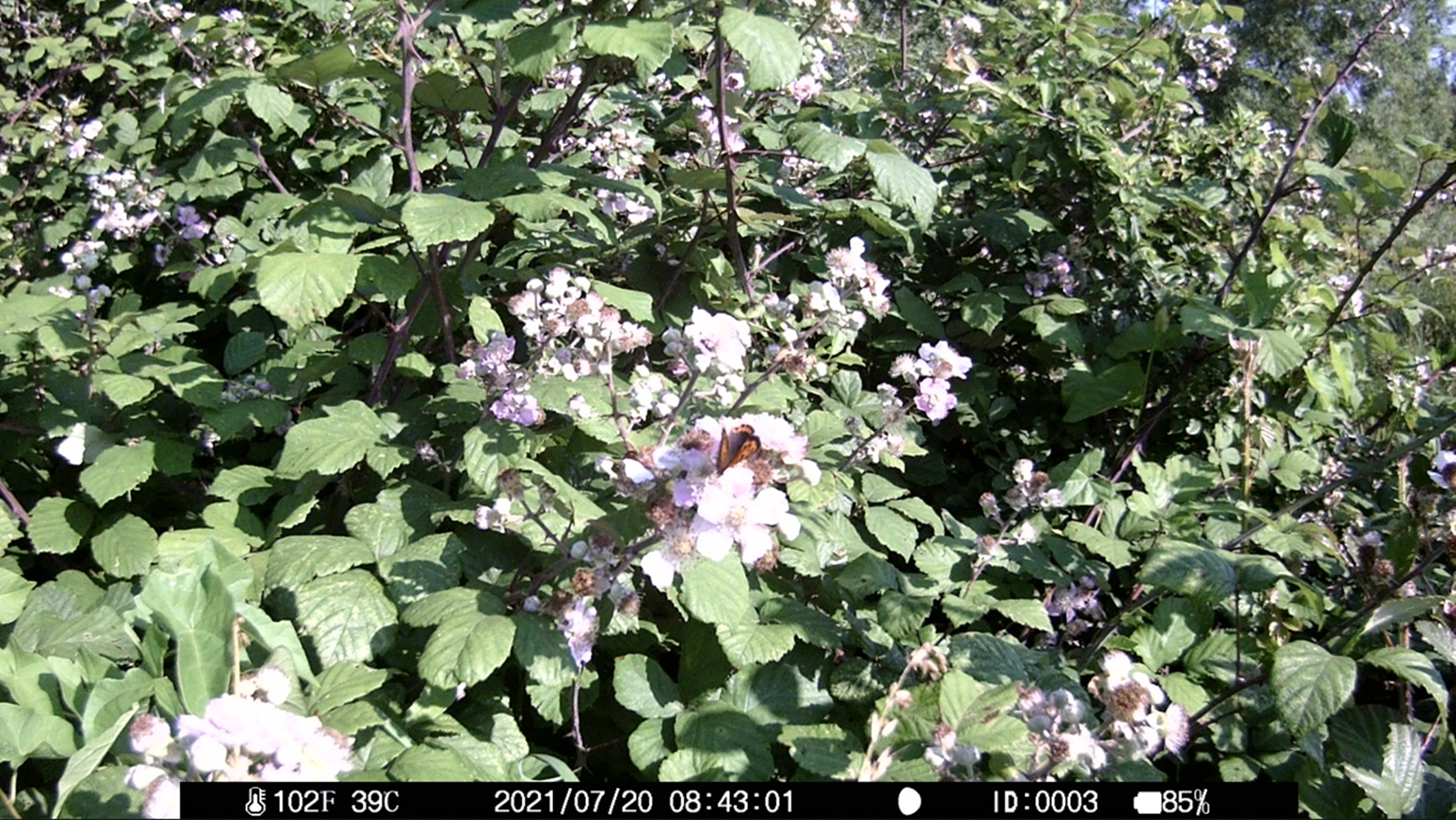

Supplement: S2 Fig — (TIF) [file pone.0281810.s002.tif]

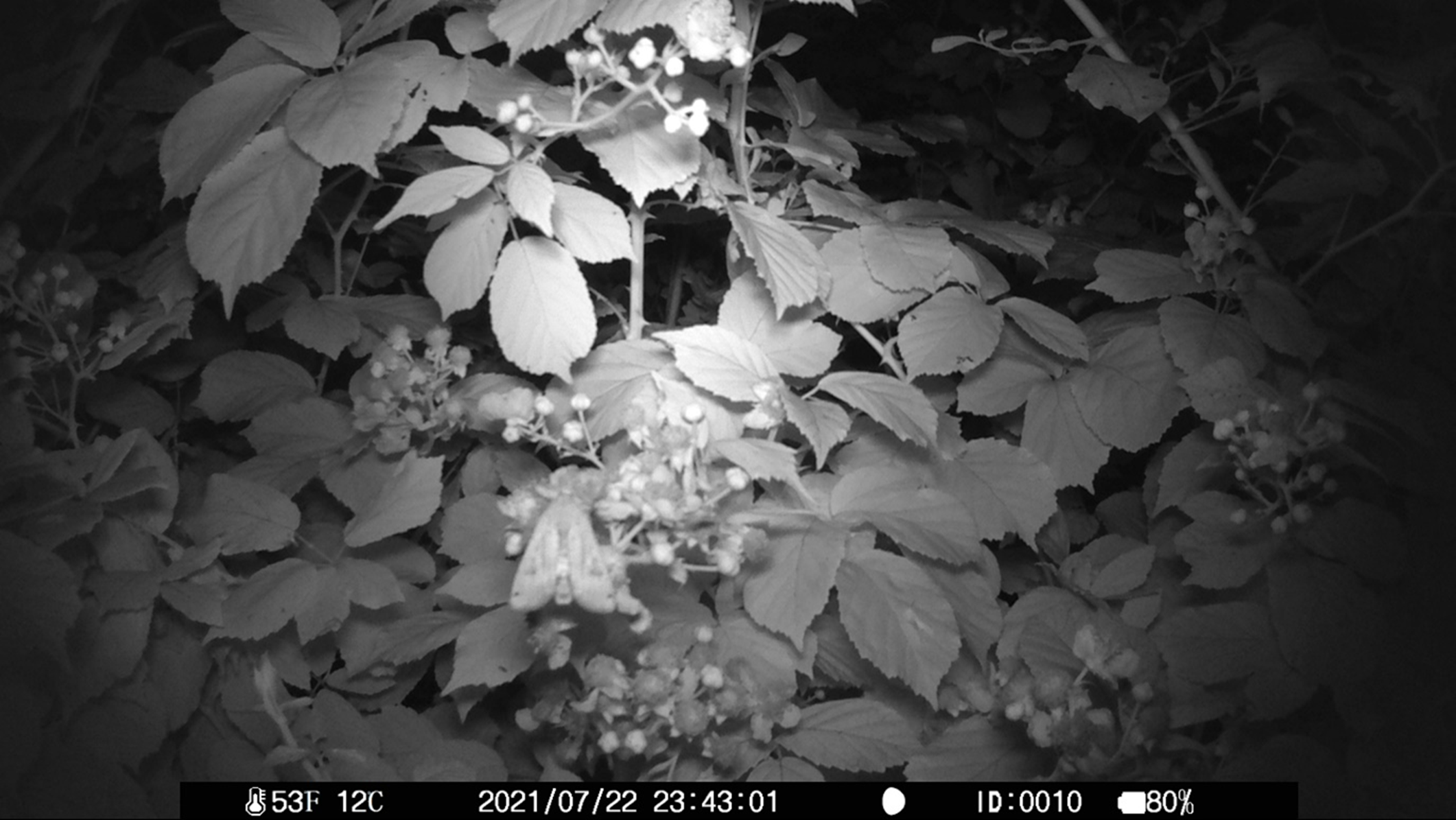

Supplement: S3 Fig — (TIF) [file pone.0281810.s003.tif]

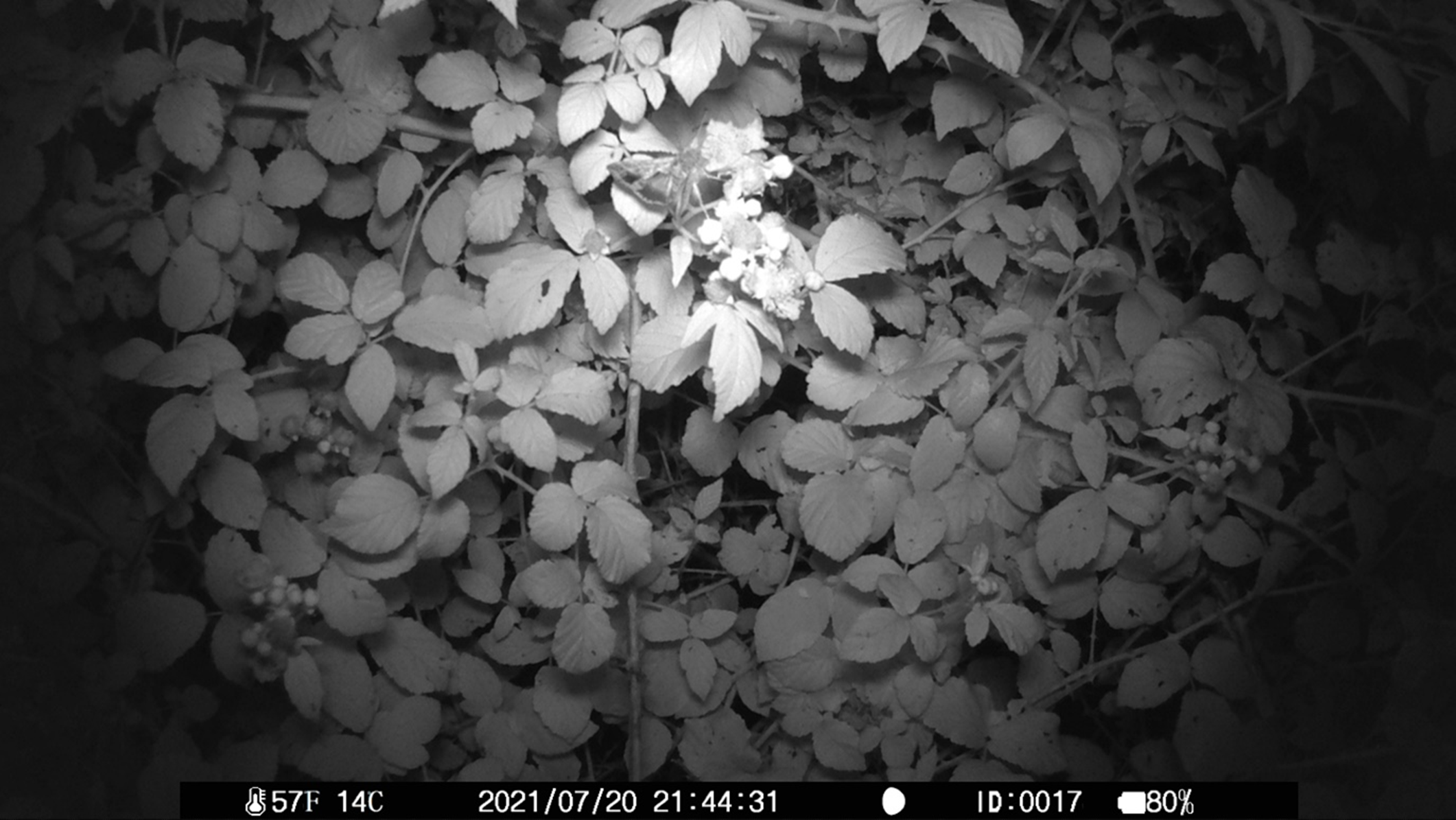

Supplement: S4 Fig — (TIF) [file pone.0281810.s004.tif]
