# Supplementary material for: Marvellous moths! pollen deposition rate of bramble (Rubus futicosus L. agg.) is greater at night than day
Source: PLoS One. 2023 Mar 29;18(3):e0281810. doi: 10.1371/journal.pone.0281810 (PMC10057810; doi:10.1371/journal.pone.0281810)
Supplement: S1 Table — (DOCX) [file pone.0281810.s007.docx]

**S1 Table.**

| **Site number** | **Sampling period** | **Mean daily temperature (°C)** | **Mean nightly temperature (°C)** | **Mean daily rainfall (mm)** |
| --- | --- | --- | --- | --- |
| **1** | 5/7/21 - 9/7/21 | 17.4 | 14.2 | 0.9 |
| **2** |  |  |  |  |
| **3** | 12/7/21 - 16/7/21 | 20.3 | 15.8 | <0.1 |
| **4** |  |  |  |  |
| **5** |  |  |  |  |
| **6** |  |  |  |  |
| **7** | 19/7/21 - 23/7/21 | 23.1 | 15.9 | <0.1 |
| **8** |  |  |  |  |
| **9** |  |  |  |  |
| **10** |  |  |  |  |
