# Supplementary material for: Marvellous moths! pollen deposition rate of bramble (Rubus futicosus L. agg.) is greater at night than day
Source: PLoS One. 2023 Mar 29;18(3):e0281810. doi: 10.1371/journal.pone.0281810 (PMC10057810; doi:10.1371/journal.pone.0281810)
Supplement: S2 Table — (DOCX) [file pone.0281810.s008.docx]

**S2 Table.**

| **Date** | **Sunrise time** | **Sunset time** |
| --- | --- | --- |
| **05/07/2021** | 04:54 | 21:17 |
| **06/07/2021** | 04:55 | 21:16 |
| **07/07/2021** | 04:56 | 21:16 |
| **08/07/2021** | 04:57 | 21:15 |
| **09/07/2021** | 04:58 | 21:15 |
| **12/07/2021** | 05:01 | 21:12 |
| **13/07/2021** | 05:02 | 21:11 |
| **14/07/2021** | 05:03 | 21:10 |
| **15/07/2021** | 05:04 | 21:09 |
| **16/07/2021** | 05:05 | 21:08 |
| **19/07/2021** | 05:09 | 21:05 |
| **20/07/2021** | 05:10 | 21:04 |
| **21/07/2021** | 05:12 | 21:03 |
| **22/07/2021** | 05:13 | 21:01 |
| **23/07/2021** | 05:14 | 21:00 |
